# A single synthetic lipid antigen for improved serological diagnosis of Buruli ulcer

**DOI:** 10.5588/pha.23.0038

**Published:** 2023-12-07

**Authors:** J. Hacking, V. V. Gwenin, R. J. Dacombe, M. S. Baird, M. Frimpong, R. O. Phillips, C. D. Gwenin

**Affiliations:** 1 School of Natural Sciences, Bangor University, Bangor, Gwynedd, Wales; 2 Liverpool School of Tropical Medicine, Liverpool, UK; 3 Kumasi Centre for Collaborative Research in Tropical Medicine, Kwame Nkrumah University of Science and Technology, Kumasi; 4 School of Medicine and Dentistry, Kwame Nkrumah University of Science and Technology, Kumasi, Ghana; 5 School of Applied Sciences, Division of Health Sciences, Abertay University, Dundee, Scotland, UK

**Keywords:** BU, point-of-care diagnosis, lipid antigen, TDM, cord factor, mycolic acid

## Abstract

**SETTING:**

The diagnosis of Buruli ulcer (BU) is frequently made by experienced health workers in rural regions. This leads to long turnaround times to confirm the diagnosis as it requires specialised laboratory infrastructure to perform confirmatory testing.

**BACKGROUND:**

Given the lack of success with protein antigens to detect BU in human sera, the aim of this study was to evaluate a range of single synthetic lipid antigens using an enzyme-linked immunosorbent assay (ELISA). The ELISA system used was initially developed to detect TB using single synthetic lipid antigens.

**METHODS:**

Thirty polymerase chain reaction (PCR) positive BU samples and 30 PCR-negative healthy contact samples collected from Asante Akim North and Ahafo Ano North Districts, Ghana, that are endemic for BU between 2013 and 2016 were used to evaluate the synthetic lipid antigen ELISA. A Quantikine ELISA was also conducted on a randomly blinded sub-set of 30 samples.

**RESULTS:**

The synthetic lipid ELISA evaluated here outperforms all other ELISA tests using protein antigens to detect BU to date and has shown potential as a fast (2 h) test for BU which may be adapted for use at the point of care. A sensitivity of 63% and specificity of 80% was observed for 30 BU-positive and 30 BU-negative samples, with significantly reduced interleukin-8 (IL-8) levels in a subset of patients with BU.

**CONCLUSION:**

A single lipid was shown for the first time to have the ability to distinguish between PCR-positive BU and negative sera using ELISA. The low lipid antibody load detected may be a result of immune suppression caused by the presence of mycolactone in patients with BU, given that levels of IL-8 were significantly reduced in patients with BU compared to the control serum samples.

Buruli ulcer (BU) was initially documented as an unusual type of skin ulcer in an Australian farming community in 1935, with the identification of the responsible mycobacterium, *Mycobacterium ulcerans *(MU), in 1948. In the 1960s, many cases emerged in Buruli County, Uganda, leading to the name ‘Buruli Ulcer’.^1^,^2^ The WHO recognised BU as a neglected tropical disease (NTD) in 1998.^3^ Passive surveillance for BU has been in place in Ghana since 1998. Given that BU predominantly affects rural communities and is accompanied by the stigma associated with the disease, this is likely to lead to systematic underreporting within a passive reporting system. A total of 106 cases were reported to the WHO for Ghana in 2022 (which indicates a prevalence below 1/100,000 population). BU has been reported in over 33 countries across ­Africa, South America and the Western Pacific, with nearly half of the African patients being children ­under 15.^4^,^5^

Traditionally, surgery was the primary treatment for BU, often involving hospitalisation and potential amputations.^6^,^7^ Since the turn of the century, there has been a significant step forward in BU treatment, initially with a combination of rifampicin and streptomycin,^2^ and now with a combination of rifampicin and clarithromycin being used for 8 weeks as opposed to surgery,^8^ regardless of disease stage.^5^

Early detection and effective treatment are crucial due to the absence of a vaccine and limited understanding of transmission modes.^9^ Developing a rapid, affordable, point-of-care (POC) test is essential, as current confirmation methods rely on laboratory tests for acid-fast bacilli (AFB) detection through direct microscopy, culture, histopathology or polymerase chain reaction (PCR) targeting the insertion sequence (IS) *2404*.^6^,^10^ Validated in 2010,^11^ PCR serves as the gold standard for BU detection, offering high sensitivity (92–95%) within 48 h but requiring substantial re­­­sources.^6^,^9^ Direct microscopy, the only low-cost POC test, exhibits limited specificity and sensitivity (40–60% or 80%, depending on the laboratory).^7^,^9^,^11^

Previously protein antigens have been used in the development of BU serodiagnostic tests with little success due to problems with antigenic cross-reactivity and the differentiation of antibody profiles at various stages of the disease.^11^

In a study by Dobos et al., 71.8% of 39 patients with BU exhibited a positive skin test response to burulin (MU whole-cell lysate), compared to 14% of 21 healthy controls.^12^ Of these patients with BU, 28.2% also showed a strong response to purified protein derivative (PPD) and burulin, indicating potential unreliability in BU/TB-endemic regions with widespread bacilli Calmette-Guérin (BCG) vaccination.^12^ These findings were corroborated by Pidot et al., who noted that most patients with BU reacted to both burulin and PPD,^11^ primarily due to antigenic cross-reactivity among environmental mycobacteria.^5^

## OBJECTIVE

This study aimed to assess synthetic lipid antigens using ELISA for BU detection in human sera, as protein antigens have been unsuccessful. Mycobacterial cells contain various mycolic acid derivatives, which can be used to characterise different mycobacteria.^13^ The ELISA system, originally designed for TB, was adapted to detect BU using synthetic lipid antigens such as alpha trehalose 6,6′-dimycolate (TDM) and trehalose 6-monomycolate (TMM). This study is the first to employ synthetic lipid antigens for BU diagnosis, potentially offering an alternative to existing tests.^14^

The study also aimed to quantify tumour necrosis factor-alpha (TNF-α), interleukin 6 (IL-6), IL-8 and IL-10 levels in serum using Quantikine (R&D Systems, Minneapolis, MN, USA) ELISA. Patients with BU were expected to exhibit lower signals than BU controls due to mycolactone, a lipid toxin produced by MU, suppressing the local inflammatory response. Mycolactone reduces the ability of dendritic cells to initiate immune responses.^15^ To note, patients with BU displayed suppressed levels of the inflammatory chemokine IL-8, which gradually increased to control levels post-antibiotic therapy.^16^,^17^ These data shed light on how mycolactone may impact antibody responses detected using ELISA.

## METHOD

### Participant serum samples

Thirty PCR-positive BU patient samples and 30 PCR-negative healthy contact samples collected between 2013 and 2016 were used to evaluate an ELISA using a synthetic lipid antigen (Table 1). Patients with BU and healthy contacts were recruited from Asante Akim North and Ahafo Ano North Districts, Ghana, where BU is endemic.

**TABLE 1:** Supplied details of the participant sera

Patient characteristics	Patients	Controls	Total
Age, years, median (range)	15.5 (5–56)	12 (5–62)	14 (5–62)
Sex, *n* (%)			
Male	11	16	27 (45)
Female	19	14	33 (55)
Lesion form, *n*			
Nodule	8		
Plaque	8		
Oedema	2		
Ulcer	12		
Category, *n*			
I	18		
II	9		
III	3		

The participant HIV status was unknown and control samples were collected from the same endemic region as the patient samples. Ethics approval was obtained from the Committee on Human Research, Publication and Ethics (CHRPE) of the Kwame Nkrumah University of Science and Technology (KNUST; Kumasi, Ghana) (approval number CHRPE/AP/229/12). All participants provided written informed consent. All study procedures conformed with the principles guiding research in human subjects as set out in the Declaration of Helsinki.^18^

### Antigens

**FIGURE 1:**
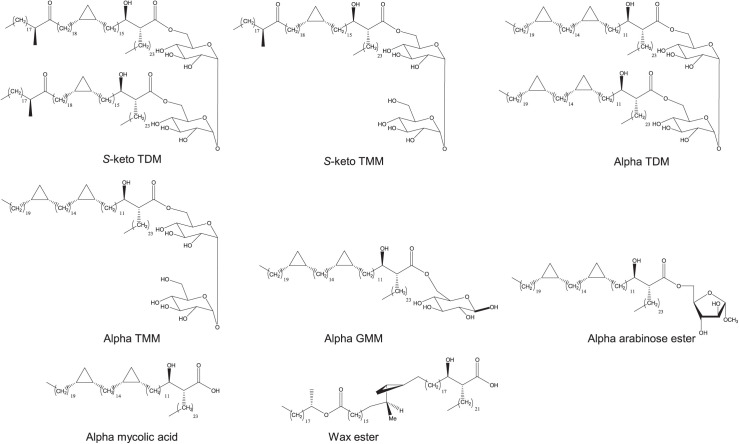
Structure of antigens tested: keto TDM, keto TMM, alpha TDM, alpha TMM, alpha GMM, alpha arabinose ester, alpha mycolic acid and wax ester from *M. avium*.^21^,^22^ TDM = alpha trehalose 6,6′-dimycolate; TMM = trehalose 6-monomycolate; GMM = glucose mycolate.

Synthetic antigens were prepared as previously described, and included pure synthetic trehalose mono- and dimycolates,^19^ a glucose mycolate (alpha GMM) (Figure 1),^20^ a non-esterified alpha mycolic acid,^23^ an arabinose ester,^24^ and a wax ester.^23^

### Enzyme-linked immunosorbent assay

The ELISA method was followed according to a previously published method,^13^ with slight modifications. Briefly, this consisted of synthetic antigens (Figure 1) being dissolved in hexane (5.6 µM) in a glass vial. Each antigen solution was vortexed for 30 sec, then pipetted into the centre of each well (50 µl/well) in a polystyrene gamma-irradiated flat-bottomed 96-well plate. Plates were left to evaporate at room temperature then covered and stored at room temperature overnight. The following day, antigen plates were incubated with 0.5% casein weight by volume (w/v) phosphate-buffered saline (PBS) (pH 7.4, 350 µl/well). After 30 min incubation at 25°C, the buffer was aspirated off using a ThermoScientific Wellwash (Waltham, MA, USA) automatic washer and the plate tapped dry. Serum was diluted 1:40 in 0.5% casein w/v PBS (pH 7.4) and added to the plate (50 µl/well). As a control for non-specific background, antigen-coated wells were incubated with buffer (instead of serum) and serum was also added to wells that were hexane-coated only (no antigen).

After 1 h of incubation at 25°C, the plate was washed three times with 0.5% casein w/v PBS using the automatic washer and tapped dry. The goat anti-human immunoglobulin G (IgG) fragment crystallisable chain specific peroxidase conjugate (2.9 µg/mL diluted in 0.5% casein w/v PBS) was added to the plate (50 µl/well) and incubated for 30 min at 25°C. The plate was washed three times and tapped dry; O-phenylenediamine (OPD; 0.2 mg/mL) and hydrogen peroxide substrate (H_2_O_2_; 0.16 mg/mL) diluted in citrate buffer (0.1 M, pH 4.5) was then added to the plate (50 µl/well). After 30 min incubation at 25°C, sulphuric acid (H_2_SO_4_; 2.5 M) was added to the plate (50 µl/well) and read at 492 nm in a Thermoscientific Multiskan-FC plate reader.

### Quantikine assay

For the Quantikine assay a sub-set of 15 negatives and 15 positives was randomly (blindly) selected from the full set.

#### Human TNF-α Quantikine kit

Assay diluent (RD1F, 50 µl) was pipetted into the centre of each well of a 96-well plate, which was pre-coated in a monoclonal antibody-specific for human TNF-α. Serum or standard (1–1,000 pg/mL TNF-α) was added (200 µl/well); the plate was then covered with an adhesive sticker and incubated for 2 h at 25°C under dark conditions.

#### Human IL-6 Quantikine kit

Assay diluent (RD1W, 100 µl) was pipetted into the centre of each well of a 96-well plate, which was pre-coated in a monoclonal antibody-specific for human IL-6. Serum or standard (0–300 pg/mL IL-6) was added (100 µl/well); plate was then covered with an adhesive sticker and incubated for 2 h at 25°C under dark conditions.

#### Human IL-8 Quantikine kit

Assay diluent (RD1-85, 100 µl) was pipetted into the centre of each well of a 96-well plate, which was pre-coated in a monoclonal antibody specific for human IL-8. Serum or standard (0–2,000 pg/mL IL-8) was added (50 µl/well); the plate was then covered with an adhesive sticker and incubated for 2 h at 25°C under dark conditions.

#### Human IL-10 Quantikine kit

Assay diluent (RD1W, 50 µl) was pipetted into the centre of each well of a 96-well plate, which was pre-coated in a monoclonal antibody specific for human IL-10. Serum or standard (0–500 pg/mL IL-10) was added (200 µl/well); plate was then covered with an adhesive sticker and incubated for 2 h at 25°C under dark conditions.

### Quantikine assay washes

The plates were then washed four times using an automatic washer and tapped dry. After adding an enzyme-linked polyclonal antibody (200 µl/well), the plate was covered and incubated for 2 h at 25°C under dark conditions. Plate was washed four times and tapped dry; the substrate solution was then added (200 µl/well) and incubated for 30 min at 25°C under dark conditions. Finally, an acid-based stop solution was added (50 µl/well), and the plates were read at 450 nm and 540 nm using a plate reader.

### Statistical analysis

The statistical analysis of the ELISA results was performed using IBM Statistical Analysis software package (IBM Corp, Armonk, NY, USA), together with MS Excel (Microsoft, Redmond, WA, USA) and included box plots and receiver operating curve (ROC) analysis. The area under the ROC and the standard error was determined at 95% confidence levels using a cut-off value selected from the ROC curve that gives specificity greater than 95%. Using the raw ELISA data at 492 nm, an optical density greater than the negative control value plus 3 standard deviations, was also selected as the cut-off value to maximise sensitivity and specificity. The accuracy was calculated in addition to sensitivity and specificity.

For the Quantikine results, the optical density was measured at 450 nm using a microplate reader and corrected by subtracting the readings at 540 nm to account for optical imperfections in the plate. All standards and serum samples were run in duplicate, with the average zero standard optical density being subtracted from each averaged optical density reading. A standard curve was used to determine the concentration of each cytokine in the ­serum sample.

## RESULTS

### Synthetic lipid enzyme-linked immunosorbent assay

For the first time, serum samples of 30 PCR-positive BU and 30 BU patient contacts were analysed using ELISA against synthetic lipid antigens (Figure 1). The antigens included TDM and TMM of an alpha-mycolic acid that was a lead antigen in the diagnosis of TB,^14^ the TDM and TMM of a keto-mycolic acid, a glucose mycolate of an alpha-mycolic acid and the free alpha-mycolic acid that did not perform as well in the diagnosis of TB,^14^ as well as an arabinose ester. Finally, a wax ester characteristic of *M. avium* and related species was used in order to determine the likely range of effective antigens.^23^ Participant details are summarised in Table 1, showing that patients and controls were well-matched. The median age of participants was similar between the two groups: patients (15.5 years, range 5–56) and controls (12 years, range 5–62). Patients presented with nodules (*n* = 8), plaque (*n* = 8), oedema (*n* = 2) and ulcer (*n* = 12) lesions, with a higher prevalence of Category 1 lesions (*n* = 18) than Category II (*n* = 9) and Category III (*n* = 3) lesions. ROCs for each antigen were generated using average absorbance at 492 nm, and optimal sensitivity and specificity were determined using a cut-off value greater than the negative control value plus 3 standard deviations (Table 2).

**TABLE 2:** ROC analysis for each antigen with Buruli ulcer sera (from 30 PCR-positive patients, 30 controls)

	Keto TDM	Keto TMM	Alpha TDM	Alpha GMM	Alpha TMM	Alpha arabinose ester	Wax ester	Alpha mycolic acid
Optimum cut-off	0.22	0.27	0.18	0.12	0.44	0.12	0.24	0.12
Optimum sensitivity, %	56.7	60	60	50	63.3	53.3	66.7	16.7
Optimum specificity, %	60	66.7	63.3	53.3	80	53.3	63.3	93.3
Accuracy	0.58	0.63	0.62	0.52	0.72	0.53	0.65	0.55
Cut-off at >95% specificity from ROC	0.49	0.6	0.36	0.21	0.97	0.23	0.56	0.33
Sensitivity from ROC at >95% specificity, %	20	23.3	16.7	13.3	23.3	10	6.7	13.3
ROC area under curve	0.62	0.64	0.66	0.53	0.72	0.57	0.66	0.4
Standard error	0.07	0.07	0.07	0.08	0.07	0.08	0.07	0.07

* The standard error of the area was determined at a 95% confidence level, and the ROC curve was used to select a cut-off value, ensuring 95% specificity. Optimal sensitivity, specificity and accuracy values for each antigen were also calculated.

ROC = receiver operating curve; PCR = polymerase chain reaction; TDM = alpha trehalose 6,6′-dimycolate; TMM = trehalose 6-monomycolate; GMM = glucose mycolate.

**FIGURE 2:**
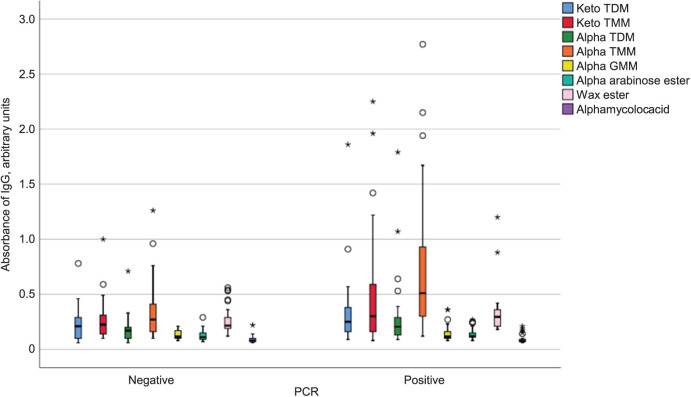
Box and whisker plots for Buruli ulcer sera samples in ELISA with keto TDM, keto TMM, alpha TDM, alpha TMM, alpha GMM, alpha arabinose ester, alpha mycolic acid, wax ester from M. avium. The data was analysed at the 95% confidence interval and the horizontal line inside the boxes indicate the median of the data points, and the boxes include data points which fall within the 1^st^ and 3^rd^ quartiles of the data set. The whiskers include the data points which fall within 1.5 of the interquartile range of both the upper and lower quartiles. Stars and circles represent outliers, with circles being the out values and stars being the extreme far out values. IgG = immunoglobulin G; TDM = alpha trehalose 6,6′-dimycolate; TMM = trehalose 6-monomycolate; GMM = glucose mycolate; PCR = polymerase chain reaction; ELISA = enzyme-linked immunosorbent assay.

The alpha-TMM antigen showed the best performance, achieving 63% sensitivity and 80% specificity with an accuracy of 0.72. Other synthetic lipid antigens had weak IgG antibody signals, except for keto TMM (Figure 2). ROC curve results (Table 1) also revealed limited differences between patient and control sera from the same endemic region. This aligns with findings by Pidot et al., who used seven purified proteins (MUP045, MUP057, MUL_0513, *Hsp65* and polyketide synthase domains enoylreductase (ER), acyltransferase (AT) propionate and ketoreductase (KR-A), and observed a low antibody response.^11^ Alpha TMM stood out as the most distinguishing antigen, offering 63% sensitivity and 80% specificity for BU-positive sera.

### ELISA response for Buruli ulcer sera to TNF-α, IL-6, IL-8 and IL-10

**FIGURE 3:**
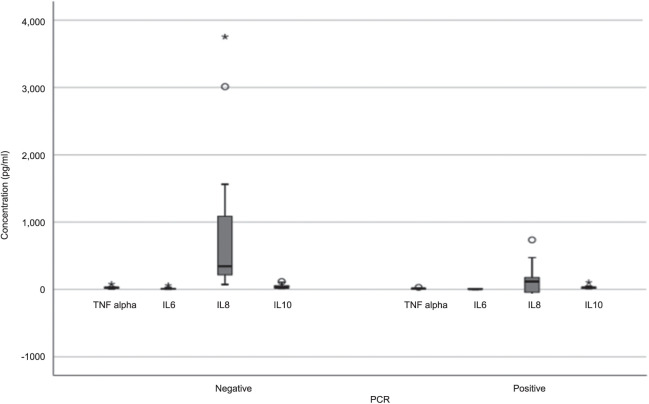
Box and whisker plots for the concentration pg/mL of TNF-α, IL-6, IL-8 and IL-10 in Buruli ulcer sera samples. The data was analysed at a 95% confidence interval and the horizontal line inside the boxes indicate the median of the data points, and the boxes include data points which fall within the 1^st^ and 3^rd^ quartiles of the data set. The whiskers include the data points which fall within 1.5 of the interquartile range of both the upper and lower quartiles. Stars and circles represent outliers, with circles being the out values and stars being the extreme far out values. TNF-α = tumour necrosis factor alpha; IL = interleukin; PCR = polymerase chain reaction; ROC = receiver operating curve.

The second aim of this study was to investigate if the low antibody levels detected in the serum of patients with BU on ELISA could be attributed to a weakened immune system, given that their HIV status was unknown. We measured TNF-α, IL-6, IL-8 and IL-10 Quantikine responses in 15 PCR-positive BU patient sera and 15 PCR-negative control sera using standard curves generated from test kit standards. Figure 3 illustrates the calculated concentrations of TNF-α, IL-6, IL-8 and IL-10 in each serum sample.

Figure 3 shows that the concentration of TNF-α, IL-6 and IL-10 detected was comparatively low in both the control and BU patient samples, whereas IL-8 had a significantly greater response with the BU control sera. The decrease in IL-8 seen in patients with BU compared to controls was consistent with findings reported by Phillips et al.,^17^ in that IL-8 is suppressed to some extent in BU cases. Further testing on patient samples after completion of antibiotic therapy would likely show a rise in IL-8 to the levels of control participants, but these serum samples were unavailable for testing in this study.

Despite a low antibody response, the synthetic lipid ELISA test surpassed all previous protein antigen-based ELISA tests for BU detection. It showed promise as a rapid (2-h) POC test. In a study with 30 BU-positive and 30 BU-negative samples from the same region, it demonstrated a sensitivity of 63% and specificity of 80% with alpha TMM (not TDM or GMM). However, there was no significant difference in IgG antibody response to seven specific proteins when comparing patient and control sera from the same region.^3^ It is important to acknowledge the limited sample size, and further research is needed, especially with different classes of mycolic acid like those with a trans-alkene at the proximal position^24^ and alternative secondary antibodies.

## CONCLUSION

In summary, a single lipid alpha TMM demonstrated 63% sensitivity and 80% specificity in distinguishing PCR-positive and ­negative BU sera on ELISA. However, the flow-through assay utilising alpha TMM lipid antigen proved unsuitable and cost-ineffective for POC BU detection due to the low lipid antibody levels in PCR-positive patients and the extent of signal amplification ­required. This reduced antibody presence may result from mycolactone-induced immune suppression in patients with BU, as indicated by significantly lower IL-8 levels, compared to control sera. Despite the low antibody response, the synthetic lipid ELISA outperforms other protein antigen-based ELISA tests for BU detection and holds promise as a rapid (2-h) POC test for the condition. It is clear that this represents only an initial study, with a modest serum sample set and a sub-set of the types of mycolic acids and associated esters that are present in mycobacteria such as MU. Moreover, because such molecules are present in all mycobacteria, additional work in necessary to exclude completely the possibility that some of the positive responses observed were caused by co-infection by other mycobacteria not excluded in the sample set. By combining a lipid ELISA and IL-8 levels in a linked assay, the risk of such interference is likely to be reduced.
